# Meet the New Health Care Gatekeeper: Your Wearable

**DOI:** 10.2196/101881

**Published:** 2026-05-29

**Authors:** Blythe Karow

**Keywords:** artificial intelligence, wearable electronic devices, remote sensing technology, delivery of health care, integrated, patient referral, reimbursement mechanisms, health policy, legislation, medical, antitrust laws, consumer health technology, clinical decision pathways, data ownership, platform regulation, commercial determinants of health

## Abstract

Consumer wearable platforms are increasingly moving into the health care space. In this *News and Perspectives* article, JMIR Correspondent and MedTech expert Blythe Karow reports on the potential implications for patient trust, policy, and regulation.

**Key Takeaways**

**Key Takeaways:**

- Consumer wearable platforms—rather than physicians—are starting to own the first conversation about health changes, influencing which specialist a patient sees and which treatments they consider.
- The policy and regulatory frameworks designed to protect patient trust were not built for a configuration in which the same company holds the user’s daily physiological data, interprets it through AI, and routes them toward a reimbursable clinical program it also bills for.
- If this consolidation continues, antitrust scrutiny becomes a legitimate question. American health care already prohibits physicians from financially benefiting when they refer patients to specific specialists or facilities. A platform that owns monitoring, interpretation, clinical routing, and reimbursement under one roof has not yet faced that same structural scrutiny.

*Blythe Karow is a strategic management consultant and founder of The Karow Advisory Group. She writes* *The Device Files**, a Substack on MedTech strategy*.

In early April, the fitness wristband company WHOOP closed a $575 million funding round at a $10+ billion valuation. The investor roster this time expanded beyond professional athletes and the expected funds to include Abbott and Mayo Clinic.

Abbott and Mayo Clinic are not consumer wellness investors. Abbott’s portfolio spans diagnostics, nutrition, and pharmaceuticals, while Mayo is one of the most credentialed clinical institutions in the country. Their presence on the cap table was a signal, for anyone paying attention, that wearables have stopped being a consumer tech play.

The next week, WHOOP made an announcement that started to clearly define that signal. A newly created affiliate called WHOOP Physician Services, PC, was selected into the first cohort of Medicare’s new ACCESS program, an outcome-based chronic care model launching in July. WHOOP, a company that spent most of 2025 in a public dispute with the Food and Drug Administration over whether its blood pressure feature qualified as a medical device, is now a Medicare-enrolled health care provider.

WHOOP’s recent strategic moves are giving us a clear picture of where the American (and maybe global) health care landscape is likely heading—and fast.

## Who Owns the First Conversation?

For decades, the entry point to medical care was the primary care physician (PCP). Your PCP decided what specialists you saw, what tests you took, what treatments you tried. Insurance networks, hospital systems, specialty referrals, and pharmaceutical distribution all organized around that referral relationship. The professional and economic architecture of American health care assumed the PCP was the first conversation.

But now a completely different category of company has been building toward accumulating something no health care incumbent possesses: rapt daily user attention on continuous physiological data. Consumer wearable platforms know when their users’ sleep is fragmenting, when heart rate variability is drifting, when blood pressure trends are climbing. They often know a user’s health is changing before the user does. And they own the first conversation about what to do about it.

That conversation used to happen in an exam room. Then people started Googling their symptoms and bugging their doctor with what they found. The next step has been ChatGPT and other large language models offering interpretation of data and test results for patients; in almost a flash, this has evolved into the wearables incorporating their own artificial intelligence to communicate within their app to the user.

And you know what? Whoever owns that first conversation is the new medical gatekeeper. They influence which specialist the user first considers, which products the user first considers buying to treat the problem, which care program the user might enroll in. That gatekeeper owns the first conversation and they know the value of that ownership. That gatekeeper can monetize and shape billions of dollars of downstream spending without having to deliver most of it.

**Figure:**
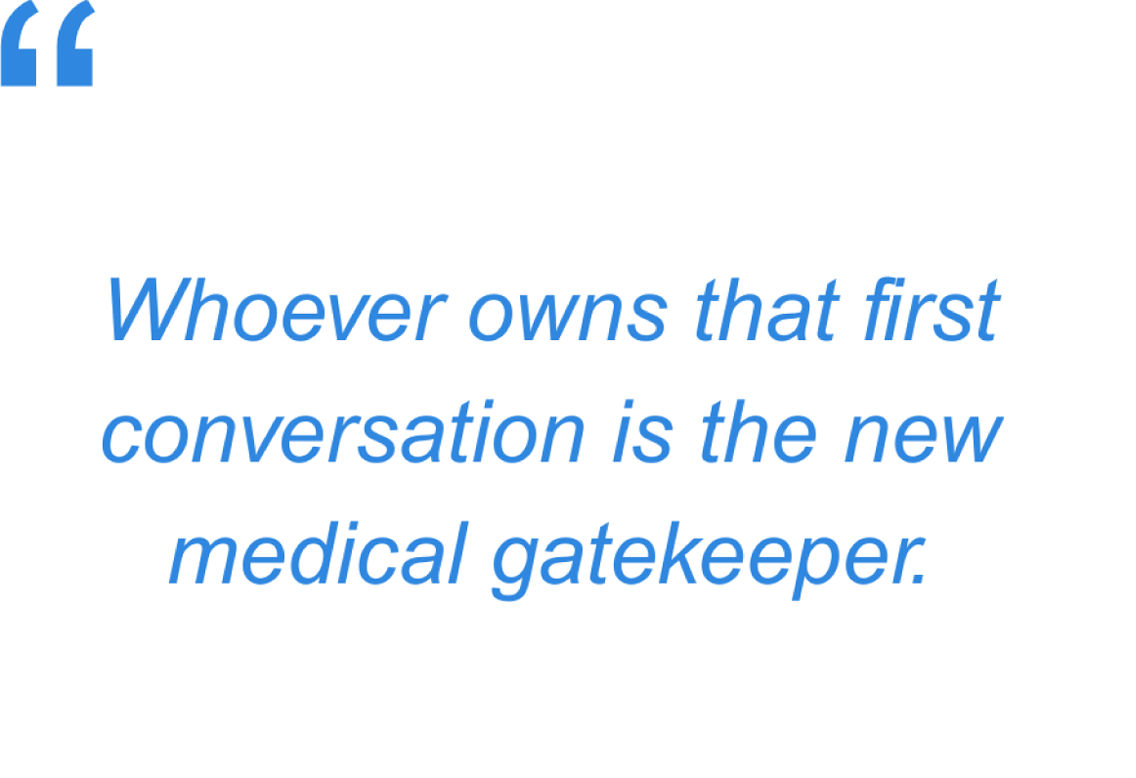


## The New Ecosystem

WHOOP is the latest visible example, but not a unique one. Oura, the smart ring maker, raised $900 million at an $11 billion valuation last October and is actively delivering on its White House commitment to integrate with Medicare’s data infrastructure, announcing electronic health record integration on the same day WHOOP announced its Medicare entry. Apple, Samsung, Withings, and Alphabet’s Verily are also actively engaging with the CMS Health Tech Ecosystem Pledge, each building out the clinical, regulatory, or reimbursement infrastructure that sits between a consumer wearable and the Medicare system. Taken together, these moves are signaling a significant change in the American health care landscape.

The wearable category is not competing to be a better fitness tracker anymore; instead, it’s competing to be the routing layer for clinical care.

This is a major shift, one with potential upsides and downsides.

The current health care system is not well served by the status quo. Doctors are overworked and spread thin and many patients aren’t proactive enough or face barriers to following up on concerning symptoms or adhering to treatment protocols. Wearables, and their apps, are well-positioned to help with a lot of that.

## A Cautionary Note

But beware who we put all of our faith in. We already know it’s probably a bad idea to give ChatGPT pictures of our lab results and have it interpret them, but many people are doing just that. Wearables companies came up in a culture of monetizing data, your data, and the one you’re wearing right now is legally selling some data about you.

Pharmaceutical and MedTech companies profit from adding patients to treatment funnels that ultimately end in purchases of their product. But they’re not controlling the funnel, monetizing the user data flowing through it, and getting to suggest which treatment a patient is routed toward. Wearable platforms are positioned to do all three at once, legally, today.

The wall between what they learn about a user as a customer and what they do with that information when they become a clinical intermediary depends entirely on how each company decides to enforce it. The concern is that wearables came up in a different culture. Their business models are built on user attention, subscription revenue, and the value of user data.

Consumer technology has spent twenty years learning how to route user attention toward outcomes that benefit the platform. When the outcome is which specialist a user sees, which prescription a user fills, or which Medicare-reimbursed program a user enrolls in, that optimization logic meets clinical judgment on terms the health care system has not previously had to confront.

The American health care system has spent decades waiting for disruption to come from better doctors, better hospitals, or better insurance. It is coming—but from a direction we’re not necessarily prepared for, and it is arriving faster than the policy and regulatory frameworks around patient trust were built to handle.

